# Supplementary material for: Fabrication of Gelatin-ZnO Nanofibers for Antibacterial Applications
Source: Materials (Basel). 2020 Dec 29;14(1):103. doi: 10.3390/ma14010103 (PMC7795140; doi:10.3390/ma14010103)
Supplement: Supplementary file 1 [file materials-14-00103-s001.pdf]

materials

MDPI

Supporting Information

Fabrication of Gelatin-ZnO Nanofibers for Antibacterial Applications

Nataliya Babayevska, Łucja Przysiecka, Grzegorz Nowaczyk, Marcin Jarek, Martin Järvekülg, Triin Kangur, Ewa Janiszewska, Stefan Jurga and Igor Iatsunskyi

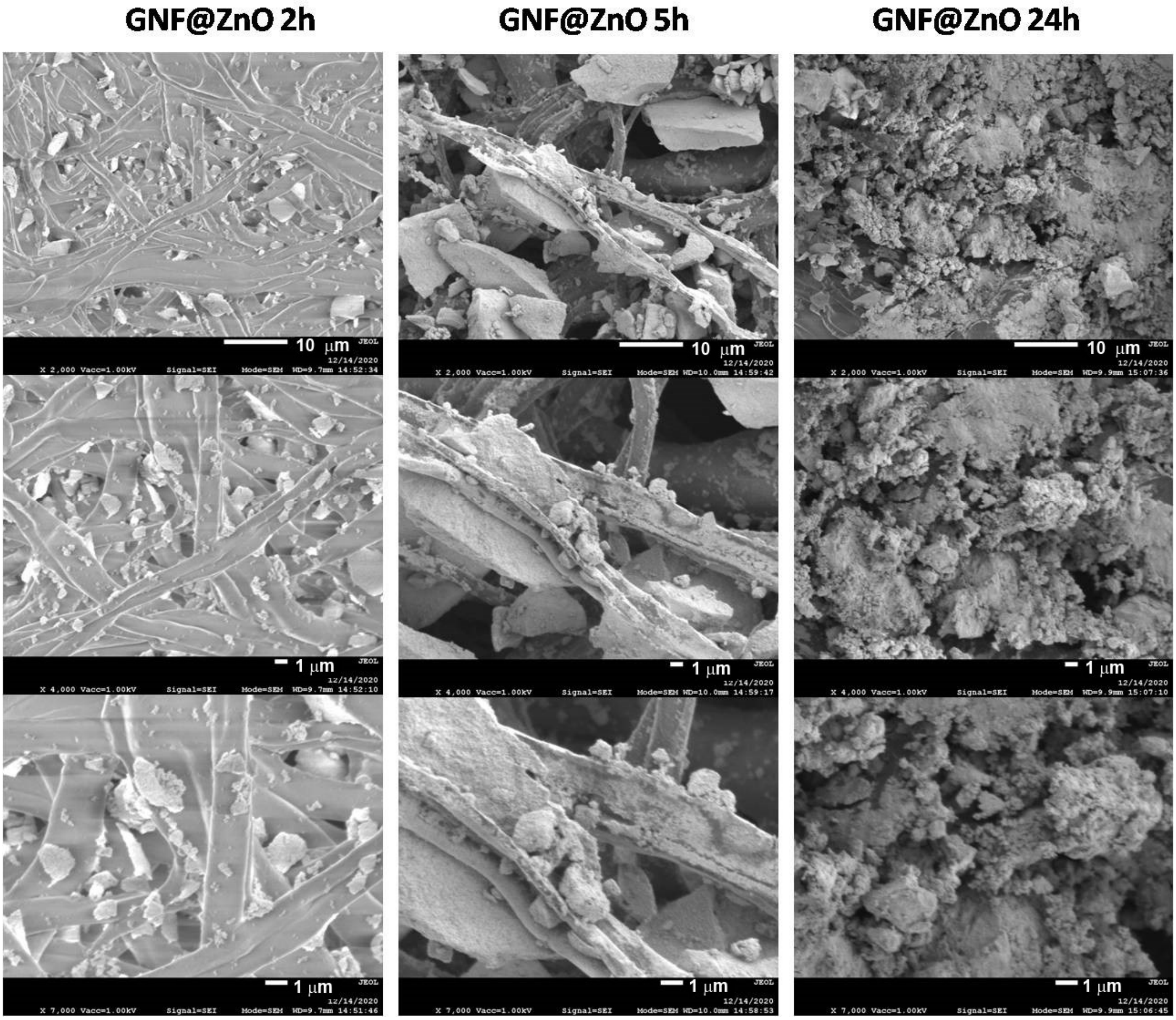

Figure S1. SEM images (magnification ×2000, ×4000 and ×7000) of GNF@ZnO 2h, GNF@ZnO 5h and GNF@ZnO 24h.

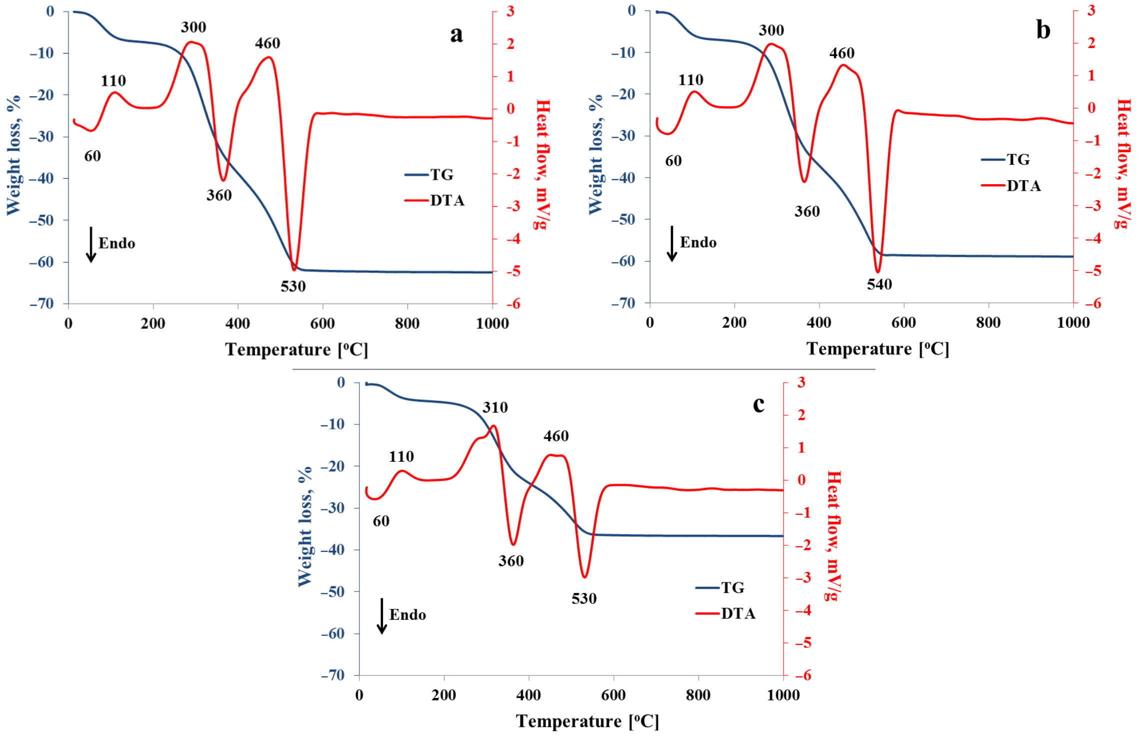

Figure S2. TG-DTA curves of GNF@ZnO 2h (a), GNF@ZnO 5h (b) and GNF@ZnO 24h (c).
